# Editorial: The Physiology of Inflammation—The Final Common Pathway to Disease

**DOI:** 10.3389/fphys.2018.01741

**Published:** 2018-12-04

**Authors:** Alexandrina Ferreira Mendes, Maria Teresa Cruz, Oreste Gualillo

**Affiliations:** ^1^Center for Neuroscience and Cell Biology, University of Coimbra, Coimbra, Portugal; ^2^Faculty of Pharmacy, University of Coimbra, Coimbra, Portugal; ^3^The NEIRID Group (Neuroendocrine Interactions in Rheumatology and Inflammatory Diseases), Servizo Galego de Saude, Santiago de Compostela, Spain; ^4^Instituto de Investigación Sanitaria de Santiago de Compostela (IDIS), Santiago University Clinical Hospital, Santiago de Compostela, Spain

**Keywords:** aging, low grade inflammation, chronic diseases, nutrition, microbiota

The 19 original and review articles included in the research topic “Physiology of Inflammation- the common pathway to disease” collectively convey an updated perspective on a variety of physiologic and pathologic conditions that trigger inflammation and how this response develops and progresses affecting tissue functions, either contributing to protect the organism or, instead, being a damaging process that disrupts homeostasis and drives most chronic diseases. While acute inflammation is usually a self-limited process, chronic inflammation, excluding that associated with autoimmune diseases, is characterized by a low-grade persistent inflammatory response that frequently is not clinically evident, but can be detected by the presence of increased levels of cytokines, chemokines, prostaglandins, nitric oxide, proteases, and other inflammatory mediators both at the tissue and plasma levels (Minihane et al., 2015). Accumulating evidence associates low grade inflammation with aging and the development and progression of most chronic diseases, from metabolic disturbances, like diabetes mellitus, obesity and the metabolic syndrome, to neurodegenerative, musculoskeletal, renal, cardiovascular diseases, and even behavioral diseases, among others (Scarpellini and Tack, 2012; Zhu et al., 2014; Nefla et al., 2016; Mihai et al., 2018; Speer et al., 2018). This low grade inflammation not only contributes to the morbidity and mortality associated with chronic diseases, as it can also impact health status in apparently healthy people. In this regard, a recent epidemiologic study involving more than 20,000 individuals found a significant increased risk in overall mortality in individuals with high low grade inflammation scores relative to those with lower scores, independently of possible confounders, including presence of chronic diseases and a number of health-related behaviors (Bonaccio et al., 2016). Moreover, low grade inflammation has also been implicated as critical determinant of chronic fatigue, either associated with cancer or constituting the chronic fatigue syndrome (Lacourt et al., 2018).

Therefore, understanding the role and mechanisms of low grade inflammation both as a contributor to those chronic diseases as well as to the overall health status is essential to provide clues for the development of innovative more efficient therapeutic and preventive strategies. The papers included in this topic contribute to these goals by providing clues for a better understanding of those roles and mechanisms, also highlighting specific issues pertaining to distinct conditions that are relevant not only to understand the underlying mechanisms, as for identification of specific targets and development of more finely-tuned therapies that address specific components and drivers of each condition. The papers included in this research topic identify relevant questions that need to be answered to achieve that goal and propose directions for future research, as well as potential solutions for, at least, some of those conditions.

One of the most recent advances in understanding the mechanisms that drive inflammation in chronic diseases is the recognition of the role of the intestinal microbiota. While much remains to be elucidated, five papers in this research topic address this subject from different perspectives. Cristiano et al. highlight the role of the intestinal microbiota and factors that modulate it, both during development and after birth, in shaping brain neuronal circuits critical for the regulation of behavior through life. The mechanisms involved are beginning to be unveiled highlighting new avenues for prevention and therapy of behavioral disorders, namely autism spectrum disorders. Adding to this, Cardoso and Empadinhas suggest that Parkinson's disease can be elicited by toxic metabolic products produced by an altered gut microbiota that, by causing mitochondrial damage, activate the neuronal innate immune system triggering inflammation which, in turn, causes neurodegeneration and, thus, can also contribute to other neurodegenerative diseases.

The articles by Meli et al; Gaifem et al., and Toussirot et al. point out aquaporins and the dietary consumption of threonine and sodium chloride as relevant modulators of intestinal inflammation whose pharmacological or nutritional modulation can impact not only intestinal diseases, like inflammatory bowel disease, but also systemic ones, including rheumatoid arthritis, and multiple sclerosis. Interestingly, such interventions affect the innate and adaptive immune systems, thus likely interfering not only with their functions in general, but also with their ability to respond to normal and altered microbiota, which together can impact on the pathogenesis of several chronic diseases.

Another emerging area of research focus on the relationships between metabolism and inflammation. In this issue, five papers address those relationships, four of them focusing on the contribution of the metabolic syndrome and other metabolic imbalances to the activation of inflammatory pathways that drive the development and progression of many musculoskeletal diseases, from osteoarthritis and sarcopenia to autoimmune diseases, like rheumatoid arthritis. The interplay between inflammation and metabolic imbalances and its impact on important homeostatic mechanisms, like the intrinsic circadian rhythm, autophagy and cell senescence, in cells of the musculoskeletal system is presented by Vinatier et al. as a critical determinant of musculoskeletal diseases and of osteoarthritis in particular. Importantly, this paper also highlights requirements of pre-clinical models to improve their validity and translational value. Collins et al. in turn, discuss the central role of skeletal muscle damage by obesity-induced inflammation in disturbing the whole musculoskeletal system and driving its associated disorders, proposing new directions for clinical risk assessment and management of patients with metabolic syndrome, as well as identifying research opportunities. Trinchese et al. evaluated the anti-inflammatory and metabolic effects of milk from different species, concluding that human and donkey milk, but not cow milk, have significant anti-inflammatory effects and improve lipid metabolism both in the liver and skeletal muscle, raising important questions as to the nutritional effects of dairy cow products. Further adding to this subject, Francisco et al. focused on the role of leptin in linking the immune system and the adipose tissue, especially in obesity, to facilitate the development of autoimmune and other inflammatory rheumatic diseases, highlighting potential therapeutic targets. Feijóo-Bandín et al. focused on a similar subject, but addressing the role of another hormone, relaxin, in modulating inflammation, and metabolism at the cardiovascular level, highlighting its potential value as a therapeutic target for cardiovascular diseases.

Still focusing on the musculoskeletal system, Pérez-Baos et al. review the mechanisms involved in systemic inflammation-associated sarcopenia, critically describing the experimental models currently in use, their triggers and mediators, with special emphasis on the role and potential as a therapeutic target of the Janus Kinase/Signal Transducer and Activator of Transcription (JAK/STAT) signaling pathway. In a correlated area, Voltarelli et al. show that subcutaneous implantation of a melanoma tumor in mice induces the production of inflammatory mediators that cause a catabolic state driving muscle loss and decreased locomotor activity. These findings contribute to explain cancer-associated cachexia, thus highlighting new therapeutic targets with relevant clinical implications. Also pertaining to cancer, the paper by Li et al. and collaborators identifies a set of genes whose lower expression in bladder cancer patients correlates with poor survival, suggesting their potential use as prognostic biomarkers. Interestingly, some of those genes are regulated by inflammatory pathways, suggesting that modulation of inflammation in bladder cancer patients may improve overall survival. Nonetheless, further studies are required to confirm this hypothesis.

Inflammation increasingly emerges as an important component of many other diseases classically envisaged as degenerative or metabolic. In this issue, three such examples are provided by the papers by Lomelí et al; Gibson et al., and Huang et al. The first shows that some inflammatory mediators, namely VCAM-1 and nitric oxide metabolites, are correlated with the degree of endothelial dysfunction found in patients with Marfan Syndrome, a connective tissue disease most frequently associated with mutations in the fibrillin-1 gene. Interestingly, that correlation seems to precede structural changes, being pointed out as a potential marker for earlier diagnosis and therapeutic intervention. Also considering the role and causes of inflammation in atherogenesis and endothelial dysfunction, although in a different context, Gibson et al. provide an up to date review on the role of individual lipids and lipid metabolites in modulating macrophage phenotype and consequently their inflammatory status. Importantly, this review identifies causes of variability in different studies on the role of lipids on macrophage functions and phenotype directly impacting endothelial dysfunction and proposes new experimental approaches to minimize this variability and improve the translational value of resulting knowledge. Finally, Huang et al. provide evidence for a crucial role of macrophages in the development of hypertensive renal injury and fibrosis which in turn further aggravates hypertension. Renal macrophages thus emerge as potential targets for the development of novel therapies to reduce hypertensive renal injury, which may also contribute to control hypertension itself.

Starting from a different point of view, but likewise aiming at identifying strategies to reduce inflammation, Guillén et al. show that conditioned medium from human adipose tissue-derived mesenchymal stem cells (ASC) has anti-inflammatory properties reducing the production of inflammatory mediators and promoting a non-inflammatory phenotype of human macrophages. Further studies to determine whether ASC from obese patients retain these anti-inflammatory properties are needed, in as much as studies to demonstrate which component(s) of the ASC secretome are endowed with these properties and their potential therapeutic efficacy in inflammation-associated diseases.

Approaching another critical component of inflammation, the article by Alonso-Pérez et al. focuses on the role of Toll-Like Receptors (TLR), particularly TLR4, in modulating anabolic and catabolic processes, many of which are activated by inflammatory mediators, like TLR agonists, in osteoblasts, osteocytes, and mesenchymal stem cells. Finally, the paper by Garcia-Rodriguez et al. further explores this subject by reviewing current knowledge on the role of TLRs on the chronic low-grade inflammation and osteogenic responses associated with calcific aortic valve disease. In this regard, the similarities between modulation of bone and valve interstitial cell functions by endogenous TLR agonists are striking, suggesting that inhibition of inflammatory responses through modulation of these receptors may have multiple effects and therapeutic applications.

With this brief summary, we expect to give the readers a broad perspective on the subjects covered by this research topic highlighting the role of inflammation in health and a variety of disease conditions, the underlying mechanisms and the targets more promising for therapy. Moreover, we also attempted to summarize the most relevant and innovative ideas that may help shape future research. Nonetheless, many other relevant and emerging aspects could have been focused. Among those, the interplay between the circadian rhythm and inflammation and the mutual regulation of clock and inflammatory/anti-inflammatory genes, both in peripheral organs and tissues and in the hypothalamic suprachiasmatic nucleus, the central circadian clock regulator, are critically involved in disease initiation and progression (Geiger et al., 2015; Man et al., 2016) and can have profound effects in the efficacy and safety of therapeutic interventions for many chronic diseases. Elucidating those interactions and their consequences is thus an emerging and exciting area of research with a foreseeable huge impact on disease management.

We hope the readers will enjoy reading these papers as much as we enjoyed editing them and we sincerely thank all contributors, authors and reviewers, for their dynamic participation and commitment that made possible this outstanding research topic.

## Author Contributions

AM drafted the manuscript. AM, TC, and OG commented on the manuscript and approved the final version.

### Conflict of Interest Statement

The authors declare that the research was conducted in the absence of any commercial or financial relationships that could be construed as a potential conflict of interest.
